# Management and outcomes of culture-negative periprosthetic joint infection: a systematic review and meta-analysis

**DOI:** 10.5194/jbji-11-413-2026

**Published:** 2026-07-14

**Authors:** Farzad Pourghazi, Seyed Mohammad Amin Alavi, Takahiro Matsuo, Georges Baaklini, Matthew P. Abdel, Aaron J. Tande, Elie F. Berbari

**Affiliations:** 1 Division of Public Health, Infectious Diseases and Occupational Medicine, Department of Medicine, Mayo Clinic College of Medicine and Science, Mayo Clinic, Rochester, MN, USA; 2 Faculty of Medicine, Ahvaz Jundishapur University of Medical Sciences, Ahvaz, 6135715753, Iran; 3 Department of Infectious Diseases, Infection Control, and Employee Health, The University of Texas MD Anderson Cancer Center, Houston, TX, USA; 4 Faculty of medicine, Saint Joseph University, Beirut, Lebanon; 5 Department of Orthopedic Surgery, Mayo Clinic, Rochester, Minnesota, USA

## Abstract

**Introduction**: Culture-negative periprosthetic joint infection (CN-PJI) remains a major diagnostic and therapeutic challenge because antimicrobial and surgical management are often empirical. **Methods**: We performed a systematic review and meta-analysis to evaluate treatment strategies and outcomes of CN-PJI. MEDLINE and Scopus were searched through 3 July 2025, and 27 cohort studies including 1399 CN-PJI patients were included. **Results**: Random-effect meta-analyses showed a pooled treatment success proportion of 83.6 % (95 % CI, 78.2–87.9), a pooled failure proportion of 17.2 % (95 % CI, 12.6–22.9), and a pooled mortality proportion of 2.4 % (95 % CI, 1.3–4.6). Subgroup analyses demonstrated lower success and higher failure with debridement, antibiotics, and implant retention compared with one-stage and two-stage exchange arthroplasty. Hip CN-PJI showed higher reported success than knee CN-PJI. Antimicrobial regimens were highly heterogeneous and were not suitable for pooled analysis. **Conclusion**: Overall, CN-PJI may have favorable outcomes, particularly with exchange arthroplasty, but prospective studies with standardized definitions and treatment reporting are needed.

## 1 Introduction

Total joint arthroplasty (TJA) is one of the most commonly performed orthopedic procedures worldwide (Sloan et al., 2018). Although rare, periprosthetic joint infection (PJI) remains a serious and uniquely challenging complication compared with infections of native bone or joint tissue (Patel, 2023). PJI occurs in approximately 2 % of total joint arthroplasties, and culture-negative infections account for approximately 5 %–42 % of all PJIs (Nelson et al., 2023; Reisener and Perka, 2018; van Sloten et al., 2022). Despite synovial fluid analysis, adjunctive methods such as implant sonication and molecular assays that improve pathogen detection, and advanced techniques including next-generation sequencing (NGS), the causative organism remains unidentified in a subset of culture-negative (CN) cases (Goh and Parvizi, 2022; Goswami et al., 2022). Because the causative pathogen remains unknown in these cases, both antibiotic selection and surgical decision-making are often empirical, and clinical outcomes remain uncertain.

The literature outlines several treatment strategies for managing CN-PJI, including debridement, antimicrobials, and implant retention (DAIR), as well as one-stage and two-stage exchange arthroplasty procedures (Tirumala et al., 2021; van Den Kieboom et al., 2021). For antimicrobial management, there is currently no consensus regarding the optimal empirical antimicrobial regimen for CN-PJI (Zhou et al., 2024). Previous studies have reported that there is no statistically significant difference in terms of outcome between CN and culture-positive PJIs (Lu et al., 2024; Reisener and Perka, 2018; Ronan et al., 2024). However, Paz et al. (2021) observed that CN-PJI may present with less severe disease and may be associated with better outcomes (Paz et al., 2021).

A 2018 systematic review of CN-PJI included a limited number of studies and provided only restricted quantitative analysis of treatment strategies and outcomes. Since then, additional studies and larger cohorts have been published. The present systematic review and meta-analysis aimed to evaluate treatment strategies and outcomes of CN-PJI, with a focus on surgical approaches and antimicrobial regimens, and to provide an updated quantitative synthesis of treatment success, failure, and mortality across available studies.

## 2 Materials and methods

This systematic review and meta-analysis was designed and conducted in accordance with the latest Preferred Reporting Items for Systematic Reviews and Meta-Analyses (PRISMA) guidelines. The study followed a predefined protocol, which is available from the first or corresponding author upon reasonable request (Page et al., 2021). The full inclusion and exclusion criteria are detailed in the Appendix.

### 2.1 Information sources and search strategies

A comprehensive literature search was performed by an experienced medical librarian to identify studies on treatment strategies for CN-PJI (see the Supplement). The search combined relevant keywords with standardized indexing terms, with full details provided in the Appendix. On 3 July 2025, searches were conducted in Ovid MEDLINE and Scopus. All records were imported into Covidence systematic review software (Veritas Health Innovation, Melbourne, Australia) for reference management, and duplicates were removed before screening.

### 2.2 Study selection

**Figure 1:**
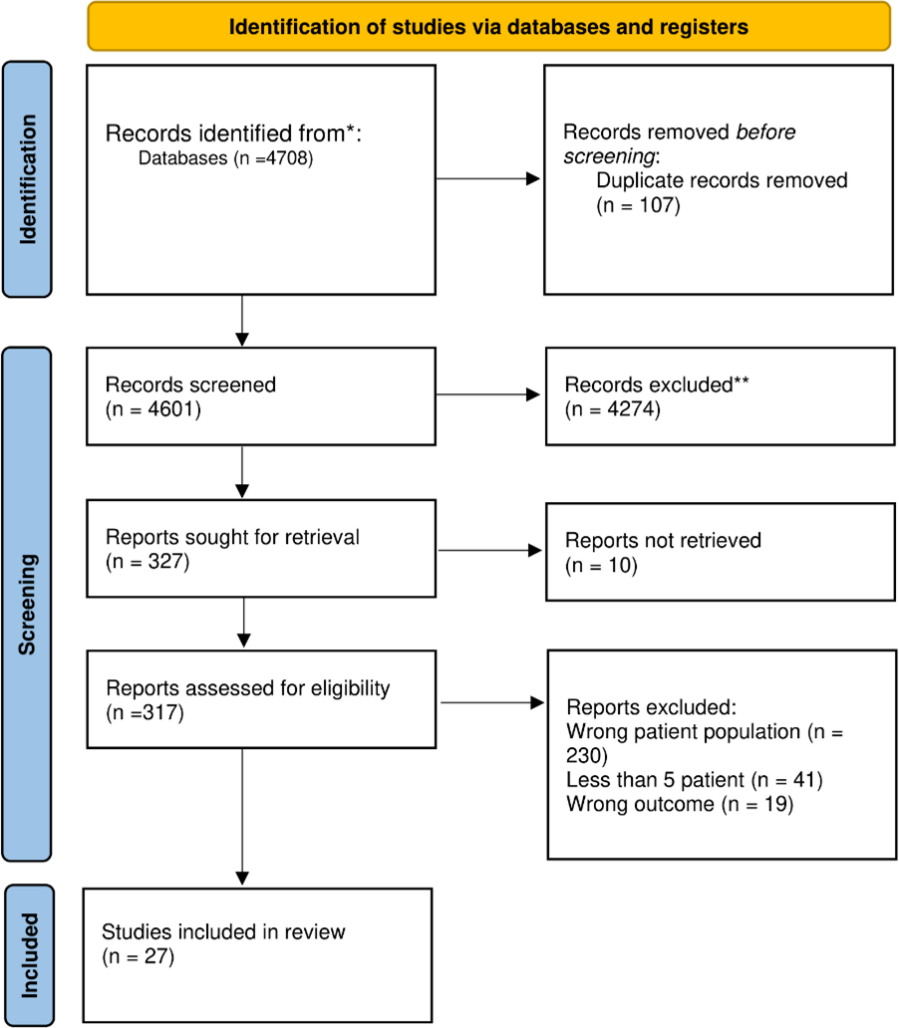
PRISMA flow chart of study selection.

Titles and abstracts, followed by full-text articles, were screened in Covidence by two independent reviewers (SMAA and FP). Discrepancies at any stage were resolved through discussion and consensus to ensure consistent study selection. Reasons for exclusion at the full-text stage are detailed in the PRISMA flow diagram (Fig. 1).

### 2.3 Data extraction

Data extraction was piloted on five studies using a standardized Microsoft Excel form to ensure clarity and consistency. After refinement based on team feedback, two reviewers (SMAA and FP) independently extracted data from all included studies into separate spreadsheets. Extracted variables are detailed in the Supplement.

Due to inconsistent reporting of antimicrobial regimens across studies, these data were summarized descriptively rather than pooled. Definitions of success, failure, and reinfection were extracted as reported by the original studies whenever available. In studies that did not provide explicit definitions, outcomes were classified based on the authors' descriptions and reported clinical criteria. Given the substantial variability in outcome definitions across the literature, success and failure outcomes were analyzed separately.

### 2.4 Risk of bias and applicability assessment

Risk of bias was assessed using the Newcastle–Ottawa Scale (NOS), which assigns up to nine stars across three domains: selection, comparability, and outcome assessment. Studies were classified as high, fair, or poor quality based on their scores across these domains according to standard NOS criteria (Wells et al., 2000). Results for quality assessment summarized in Table S1 in the Supplement.

### 2.5 Statistical analysis

All analyses were performed in R version 4.5.1 (R Foundation for Statistical Computing, Vienna, Austria) using RStudio (Posit Software, Boston, MA, USA). Because clinical and methodological heterogeneity was expected, pooled estimates were calculated using random-effect models. Meta-analysis was limited to studies including more than five patients with CN-PJI and extractable outcome data. Treatment success, failure, and mortality were pooled using random-intercept logistic regression models with logit transformation and maximum-likelihood estimation of $\tau^{2}$ . This generalized linear mixed-model approach was used because it performs well for sparse proportion data, particularly when event rates are very low or close to 0 or 1, and avoids the need for continuity corrections. Pooled proportions are reported with 95 % confidence intervals (CIs). Heterogeneity was assessed using Cochran's Q test and the $I^{2}$ statistic and was considered to be significant at Q test p<0.05 or $I^{2}>50$ %. Robustness was evaluated using outlier, leave-one-out, and influence analyses. Prespecified subgroup analyses were performed according to treatment strategy and joint type, as data allowed. Publication bias was assessed with funnel plots and Egger's regression test for outcomes with at least 10 studies; when asymmetry was detected, trim-and-fill analysis was applied.

## 3 Results

### 3.1 Search results

An electronic search of MEDLINE and Scopus retrieved 4708 papers. After removing 107 duplicates, 4601 records were screened based on titles and abstracts, and 327 full texts were sought. A total of 10 reports could not be retrieved, leaving 317 full texts reviewed for eligibility. A total of 290 were excluded. Among the excluded studies, 230 did not include CN patients in their study population, 10 included fewer than five CN patients, and 19 reported outcomes that were not eligible for this review. Finally, 27 studies (see the Appendix for a full reference list) were included in this systematic review (Fig. 1) (Berbari et al., 2007; Browning et al., 2022; Choi et al., 2013; Garabano et al., 2022; Ibrahim et al., 2018; Ji et al., 2020; Kang et al., 2018; Kim et al., 2021, 2015a, b; Li et al., 2017; Lin et al., 2025; Lu et al., 2024; Malekzadeh et al., 2010; Paz et al., 2021; Ronan et al., 2024; Santoso et al., 2018; Tan et al., 2018; Tirumala et al., 2021; van Den Kieboom et al., 2021; van Sloten et al., 2022; Wang et al., 2018; Soundarrajan et al., 2023; Vajapey et al., 2021; Watanabe et al., 2021; Xu et al., 2022; Zanna et al., 2023).

### 3.2 Study characteristics

The general characteristics of the included studies are presented in Table 1, and the findings are summarized in Table 2. All studies were cohort designs, including 26 retrospective cohorts and 1 prospective cohort, published between 2007 and 2025.

**Table 1:** General characteristics of included studies.

Author, year	Inclusion criteria	Exclusion criteria	Total population ( N of CN patients)	Female (%)	Joints	Age	Definition for diagnosis of PJI used by the authors
Berbari, 2006, USA	All patients with CN-PJI	n/a	60 (60)	28 (46.7)	Hip: 27; knee: 33	Median: 69	Other
Browning, 2022, Australia	All patients diagnosed with PJI with at least 24 months follow up	n/a	650 (55)	23 (41.8)	Hips: 18; knees: 31; shoulders: 5; elbow: 1	70.5	MSIS 2014
Choi, 2013, USA	All patients with PJI	Lost to follow-up	175 (40)	16 (40 %)	Hips: 20; knees: 20	63.9	MSIS 2011
Garabano, 2022, Argentina	Patients who underwent primary THA at our hospital and developed suspected PJI based on preoperative and intraoperative microbiologic evaluation	Positive culture	81 (12)	8 (66.7 %)	Hips: 12	73.5	NR
Ibrahim, 2018, UK	Consecutive patients who underwent two-stage revision THA for infection	Patients with acute PJI, metal-on-metal bearing surfaces, or one-stage revision	100 (50)	27 (54 %)	Hips: 50	74	NR
Ji, 2020, China	All patients with chronic PJI underwent one-stage exchange	Patients who died of unrelated causes during follow-up or had less than 2 years of follow-up	243 (51)	31 (60.8 %)	Hips: 19; knees: 32	59.3	MSIS 2011
Kang, 2018, South Korea	All patients with PJI	n/a	85 (15)	NR	Hips: 15	66.7	MSIS 2011
Kim, 2021, South Korea	Patients with chronic PJI who underwent two-stage revision TKA with antibiotic-impregnated cement spacers after primary or revision TKA	Patients with acute postoperative or acute hematogenous PJI within 1 month of symptom onset and those not treated with two-stage revision	136 (57)	43 (75.4 %)	Knees: 57	72.4	MSIS 2014

**Table 1:** Continued.

Author, year	Inclusion criteria	Exclusion criteria	Total population ( N of CN patients)	Female (%)	Joints	Age	Definition for diagnosis of PJI used by the authors
Kim, 2015, South Korea	All infected total knee arthroplasties	Of the 209 patients, 11 were lost to follow-up before 2 years, and 7 died, leaving 191 patients (191 knees) for review	191 (51)	37 (66.7 %)	Knees: 51	67.4	Other
Kim, 2015 (Journal of Orthopedics), South Korea	All patients with infected TKA	11 were lost to follow-up before 2 years, and 7 died, leaving 242 patients (242 knees) for review	242 (102)	74 (72.5)	Knees: 102	67.4	MSIS 2011
Li, 2017, China	Suspicious infected TKA revision patients	Patients with rheumatoid arthritis, ankylosing spondylitis, or traumatic arthritis	145 (18)	NR	Knees: 18	NR	ICM 2014
Lin, 2025, China	Patients diagnosed with PJI, patients who provided informed consent, and patients with complete clinical data	Patients with malignant tumors, other active infections, CN/mNGS-negative PJI, or no mNGS testing and follow-up of less than 2 years	223 (55)	29 (52.7 %)	Hips: 27; knees: 28	68	MSIS 2011

**Table 1:** Continued.

Author, year	Inclusion criteria	Exclusion criteria	Total population ( N of CN patients)	Female (%)	Joints	Age	Definition for diagnosis of PJI used by the authors
Lu, 2024, China	Patients who underwent revision surgery	Patients with malignant tumors; infection at other body sites; aseptic loosening; incomplete two-stage revision; or alternative treatment strategies such as one-stage revision, joint fusion, or DAIR	87 (24)	16 (66.7 %)	Hips: 13; knees: 11	58.1	MSIS 2014
Malekzadeh, 2019, USA	All patients with PJI	n/a	270 (135)	64 (47 %)	Hips: 68; knees: 67	68.9	
Paz, 2021, USA	Patients with documented prosthetic joint infection who underwent surgery during the index admission	Patients with prior prosthesis removal from the index joint, polyarticular septic arthritis, septic bursitis, osteomyelitis, or septic arthritis of the index joint within 90 d before admission	196 (48)	31 (64.6 %)	Knees: 44; hips: 4	62.1	None
Ronan, 2024, USA	Patients indicated for two-stage revision TKA who completed at least the first stage at the primary institution and had at least 1 year of follow-up	Patients treated with DAIR alone; those who underwent aseptic revision only; and those with incomplete demographic, surgical, or follow-up data	138 (40)	27 (67.5 %)	Knees: 40	61.25	MSIS 2018
Santoso, 2018, South Korea	Patients with PJI treated with two-stage revision arthroplasty	Ten patients were lost to follow-up	84 (27)	12 (44.5 %)	Hips: 27	67.4	MSIS 2011

**Table 1:** Continued.

Author, year	Inclusion criteria	Exclusion criteria	Total population ( N of CN patients)	Female (%)	Joints	Age	Definition for diagnosis of PJI used by the authors
Soundarrajan, 2023, India	Consecutive patients who underwent DAIR for acute PJI	Patients treated with DAIR for revision TKA PJI, those with late chronic infection, soft tissue compromise with sinus tract, or cement spacer placement as the first-stage procedure for PJI	36 (20)	NR	Knees: 20	NR	MSIS 2011
Tan, 2018, USA	Patients who underwent surgery for treatment of CN PJI	Patients with unavailable culture results, only one positive culture, megaprosthesis, subsequent PJI in the same joint, or follow-up <1 year	219	NR	Hip: 138; knee: 81	NR	MSIS 2014
Tirumala, 2021, USA	Consecutive patients with primary THA or TKA who underwent DAIR with modular component exchange for acute PJI	Type III/late chronic infection ( >4 weeks of symptoms), prior revision of the same joint, surgery within 3 months before DAIR, or unavailable joint aspirate culture results	149 (46)	24 (52.2 %)	Hips: 20; knees: 26	66.9	MSIS 2018
Vajapey, 2021, USA	Adult patients with PJI and at least 2 years of follow-up	History of malignancy, indeterminate alpha-defensin test, or incomplete medical records	15 (7)	3 (75 %)	Hips: 2; knees: 5	59.9	MSIS 2018 and 2014

**Table 1:** Continued.

Author, year	Inclusion criteria	Exclusion criteria	Total population ( N of CN patients)	Female (%)	Joints	Age	Definition for diagnosis of PJI used by the authors
Van Stolen, 2022, the Netherlands	Patients with hip or knee arthroplasty who underwent surgery for infection (DAIR, one-stage exchange, or two-stage exchange) with adequate intraoperative culture sampling	At least one positive culture, antibiotics before revision surgery unless stopped ≥2 weeks preoperatively, or follow-up <1 year for acute infection or <2 years for chronic infection	70 (70)	35	Hips: 18; knees: 52	NR	ICM 2014, MSIS 2018, EBJIS 2021
Wang, 2018, China	Adult patients with THA PJI and minimum follow-up of 6 months after reimplantation or 9 months after two-stage revision	Mixed treatment strategies, combined hip and knee revision, insufficient follow-up without an endpoint event, non-prosthetic hip infection, or superficial infection only	58 (19)	11 (58 %)	Hips: 19	61	MSIS 2011
Watanabe, 2021, Japan	Patients with CN-PJI	N/A	109 (27)	14 (51.86 $)	Hips: 19; knees: 8	72	ICM 2018
Van den Kieboom, 2021, USA	All consecutive patients who underwent revision THA or TKA for chronic CN PJI between 2010 and 2018	Prior revision for infection, hemiarthroplasty PJI, isolated bearing exchange, not meeting PJI criteria, or missing outcome data	140 (105)	55 (52.4 %)	Hips: 39; knees: 66	65.9	MSIS 2011 and 2018

**Table 1:** Continued.

Author, year	Inclusion criteria	Exclusion criteria	Total population ( N of CN patients)	Female (%)	Joints	Age	Definition for diagnosis of PJI used by the authors
Xu, 2022, China	Patients diagnosed with PJI by MSIS criteria who underwent surgery at our center, had regular follow-up, and had complete medical records	Exclusion criteria: patients who had immunosuppressive disease, infectious disease in other part, or malignant tumors; patients with negative culture during treatment were defined as CN PJI, and patients with pathogenic bacteria cultured in more than one sample were defined as CP PJI	77 (24)	12 (50 %)	Hips: 17; knees: 7	62.5	MSIS 2011 and 2018
Zanna, 2023, Germany	Patients with PJI diagnosed by 2018 ICM criteria who underwent one-stage septic revision with positive intraoperative cultures and at least 12 months of follow-up	Preoperative positive culture, negative intraoperative culture, or follow-up <1 year	22 (22)	6 (27.3 %)	Hips: 12; knees: 10	73.2	ICM 2018

Abbreviations: CN – culture-negative; CP – culture-positive; PJI – periprosthetic joint infection; THA – total hip arthroplasty; TKA – total knee arthroplasty; DAIR – debridement, antibiotics, and implant retention; MSIS – Musculoskeletal Infection Society; ICM – International Consensus Meeting; EBJIS – European Bone and Joint Infection Society; mNGS – metagenomic next-generation sequencing; NOS – Newcastle–Ottawa Scale; NR – not reported; n/a – not applicable; N – number.

**Table 2:** Summary of included studies reporting antimicrobial treatment strategies, surgical interventions, and clinical outcomes in culture-negative periprosthetic joint infection. Antibiotic regimens and durations are reported as described in the original studies and are presented to the extent that they were extractable. Outcomes are shown overall and, when available, according to surgical treatment strategy.

Author, year	Final N patients with CN PJI	Minimum follow-up duration (months)	Surgical intervention	Outcome
Berbari, 2006	60	60	Two-stage exchange: 34/60 DAIR: 12/60 One stage exchange: 5/60 Resection: 8/60 Amputation: 1/60	Overall failure: 10/60 Other outcomes NR separately for treatment strategies
Browning, 2022	55	24	DAIR: 30 Two-stage exchange: 13 Intervention for 15 pts NR	Overall success: 41/55 DAIR: 23/29 success Two-stage exchange: 8/11 success Outcome for one DAIR and two two-stage exchange NR
Choi, 2013	40	24	DAIR: 11/40 Implant removal: 23/40 Other primary treatment: 6/40	Overall success: 34/40 Outcome NR separately for different treatment
Garabano, 2022	12	25.5	DAIR: 8 Two-stage: 4	12/12 overall success DAIR: 8/8 success Two-stage: 4/4 success
Ibrahim, 2018	50	60	Two-stage: 50	Two-stage: 47/50 success (infection free)
Ji, 2020	51	24	One-stage exchange: 51	One-stage exchange: 46/51 success
Kang, 2018	15	60	Two-stage exchange: 15	Two-stage exchange: 15/15 success
Kim, 2021	57	2	Two-stage exchange: 57	Two-stage exchange: 38/57 success
Kim, 2015	51	60	DAIR: 28 Two-stage exchange: 23	Overall success: 36/51 DAIR: 17/28 success Two-stage exchange: 19/23 success
Kim, 2015 (Journal of Orthopedics)	102	72	DAIR: 56 Two-stage exchange: 46	Infection eradicated in all patients at the end of treatment, but for first treatment overall success: 70/102 DAIR: 32 success Two-stage exchange: 38 success
Li, 2017	18	12	Two-stage exchange: 18	Two-stage exchange: 15/18 success
Lin, 2025	55	24	DAIR: 7; one-stage: 16; two-stage: 32	Overall success: 48/55 Outcome NR separately for different treatment
Lu, 2024	24	24	Two-stage exchange: 24	Two-stage exchange: 23/24 success
Malekzadeh, 2019	135	24	Two-stage: 56 Resection: 34 DAIR: 18 Amputation: 5 One-stage exchange: 8 Chronic suppression: 13 No therapy: 1	Overall success: 94/135 Success and failure NR per treatment strategy

**Table 2:** Continued.

Author, year	Final N patients with CN PJI	Minimum follow up duration (months)	Surgical intervention	Outcome
Paz, 2021	48	NR	Arthroscopic surgery: 5 DAIR: 31 One-stage exchange: 3 Two-stage exchange: 5 Resection: 3	Overall CN outcome – ICU admission: 4/48 Readmission: 6/48 Deaths: 0
Ronan, 2024	40	12	Two-stage exchange: 40	Outcome reported using MSIS – Tier 1 (infection control without suppressive antibiotics): 21/40 Tier 2 (infection control with suppressive antibiotics): 4/40 Tier 3 (reoperation/spacer retention): 15/40 Tier 4 (all-cause mortality): 0/40 Overall success: 25/40
Santoso, 2018	27	12	Two-stage exchange: 27	Two-stage exchange: 25/27 success
Soundarrajan, 2023	20	36	DAIR: 20	DAIR: 14/20 success
Tan, 2018	159^*^	12	Two-stage exchange: 118; DAIR: 27; one-stage: 14	Overall success: 110/159 Two stage: 84/118 success DAIR: 15/27 success One-stage: 11/14 success,
Tirumala, 2021	46	36	DAIR: 46	DAIR: 40/46 success
Vajapey, 2021	7	4	One-stage exchange: 4 No surgery: 3	Overall outcome: success 5/7 Outcome NR separately for treatment strategies
Van den Kieboom, 2021	105	30	One-stage: 30 Two-stage exchange: 75	Overall success: 85/105 One-stage: 25 success Two-stage exchange: 60 success
Van Stolen, 2022	70	24	No-antibiotic group – DAIR: 1 One-stage exchange: 21 Two-stage exchange: 12 Antibiotic group – DAIR: 8 One-stage exchange: 6 Two-stage exchange: 22	Recurrent infection: 3/70 Other outcomes NR separately for different treatment
Wang, 2018	19	44	Two-stage exchange: 19	Two-stage exchange: 19/19 success
Watanabe, 2021	27	18	DAIR: 7 One-stage exchange: 4 Two-stage exchange: 12 Resection arthroplasty: 2/27 Observation: 2/27	Overall success: 20/27 Outcome NR separately for different treatment
Xu, 2022	24	12	DAIR: 7 One-stage exchange: 6 Two-stage: 9 Two patients did not complete the two-stage exchange treatment	Overall success: 19/22 DAIR: 6/7 success One-stage exchange: 4/6 success Two-stage exchange: 9/9 success
Zanna, 2023	22	12	One-stage exchange: 22	One-stage exchange: 20/22 success

Abbreviations: CN – culture-negative; PJI – periprosthetic joint infection; CN-PJI – culture-negative periprosthetic joint infection; N – number; AB – antibiotics; IV – intravenous; DAIR – debridement, antibiotics, and implant retention; NR – not reported; ICU – intensive care unit; MSIS – Musculoskeletal Infection Society. ^*^ A total of 60 patients were excluded from outcome analysis.

The study size ranged from 7 to 159 CN patients, with a total of 1399 patients included in the systematic review (584 hips, 809 knees, 5 shoulders, and 1 elbow). However, the number of patients included in each meta-analysis varied due to incomplete outcome reporting or non-quantifiable data across studies. Antibiotic regimens varied between studies and are summarized in Table 2 where extractable.

### 3.3 Antibiotic regimen across included studies

Antimicrobial regimens varied substantially across included studies and were often reported incompletely or were non-standardized (Table 2). Several studies did not report antibiotic details, while others provided only partial information on preoperative exposure, postoperative agents, route, or duration. Overall, antimicrobial therapy appeared to be individualized rather than protocol based. Among studies with extractable data, vancomycin-based postoperative regimens were the most reported, either alone or in combination with other agents. Cephalosporins, particularly cefazolin, ceftriaxone, ceftazidime, and cefepime, were also frequently used, and fluoroquinolones such as ciprofloxacin and levofloxacin were commonly reported as oral step-down therapy. Treatment duration also varied considerably. Because of this marked heterogeneity and incomplete reporting, antimicrobial regimens were summarized descriptively and were not suitable for quantitative synthesis (Table 2).

**Figure 2:**
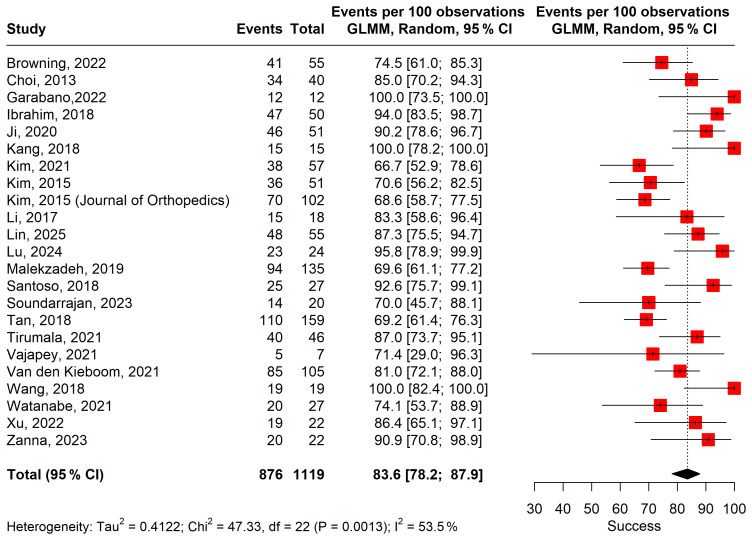
Forest plot of pooled overall treatment success. The figure shows the proportion of treatment successes reported by each included study and the overall pooled estimate using a random-effect generalized linear mixed model (GLMM).

### 3.4 Meta-analysis results

#### 3.4.1 Treatment success

A meta-analysis of 23 studies including 1119 patients and 897 treatment success events showed a pooled treatment success proportion of 83.6 % (95 % CI, 78.2–87.9) under a random-effect model (Fig. 2). Between-study heterogeneity was substantial ( $\tau^{2}=0.41$ , $I^{2}=53.5$ %, p value < 0.001).

Sensitivity analyses supported the robustness of the pooled treatment success estimate. In leave-one-out analyses under the random-effect model, omission of any single study did not materially change the pooled success proportion, which ranged from 82.6 % to 84.3 % versus an overall estimate of 83.6 %. Heterogeneity also remained similar ( $I^{2}$ , 47.1 %–55.6 %). Influence analysis suggested that four studies contributed disproportionately to between-study heterogeneity (Kim et al., 2015a, b; Malekzadeh et al., 2010; Tan et al., 2018); excluding these studies resulted in a modestly higher pooled success estimate and lower heterogeneity (see Fig. S1 in the Supplement).

Publication bias was assessed as at least 10 studies were available. Funnel plot asymmetry (Fig. S2) was confirmed by Egger's test (intercept 2.365, p<0.001 ). Trim-and-fill analysis imputed nine potentially missing studies, yielding an adjusted pooled success rate of 74.0 % (95 % CI, 67.0–80.0), compared with the primary estimate of 83.6 %, suggesting possible overestimation of success.

For subgroup analyses, some studies were excluded from the overall pool due to unavailable stratified data by treatment strategy or joint type. Pooled success differed significantly by treatment strategy ( p<0.0001 ): 73.2 % (95 % CI, 61.2–82.5; $I^{2}=52.9$ %) for DAIR, 87.4 % (95 % CI, 79.2–92.7; $I^{2}=43.4$ %) for two-stage exchange, and 86.2 % (95 % CI, 78.9–91.2; $I^{2}=0$ %) for one-stage exchange (Fig. S3).

By joint type, pooled success was 94.2 % (95 % CI, 89.2–96.9; $I^{2}=0$ %) for hip PJI and 75.8 % (95 % CI, 65.7–83.6; $I^{2}=16.8$ %) for knee PJI, with a significant subgroup difference ( p<0.001 ) (Fig. S4). This finding should be interpreted cautiously as it may reflect differences in patient selection, characteristics, or treatment practices rather than a true biological effect.

#### 3.4.2 Treatment failure

**Figure 3:**
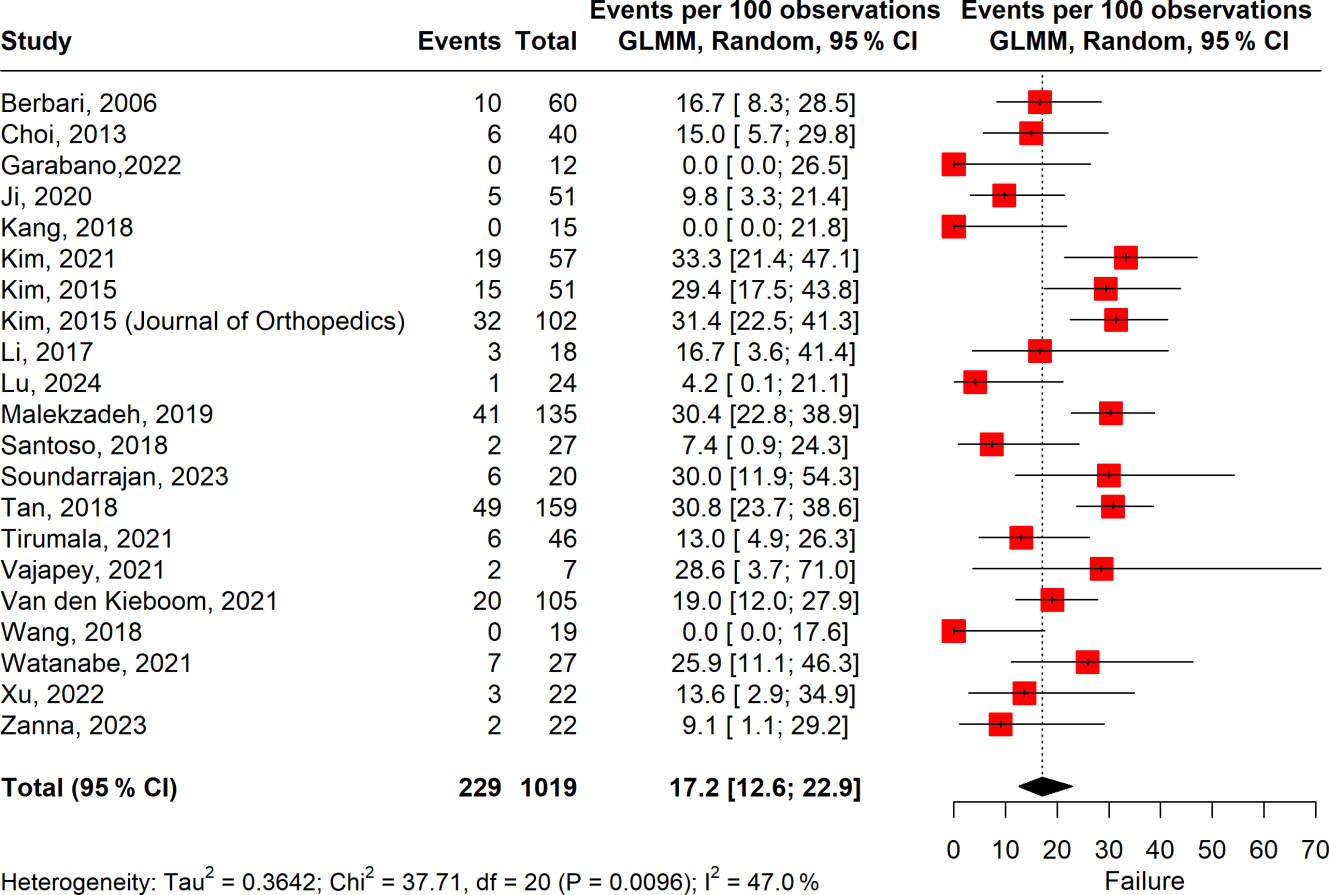
Forest plot of pooled overall treatment failure. Individual study-specific failure rates and corresponding 95 % confidence intervals are shown.

A meta-analysis of 25 studies including 1019 patients and 229 failure events yielded a pooled treatment failure proportion of 17.2 % (95 % CI, 12.6 %–22.9 %) under a random-effect model (Fig. 3). Between-study heterogeneity was moderate to substantial ( $\tau^{2}=0.36$ , $I^{2}=47$ %, p value = 0.009).

Influence analysis (Fig. S5) showed that Tan et al. (2018) had the greatest influence on the pooled failure estimate and between-study heterogeneity (Tan et al., 2018), followed by Malekzadeh et al. (2010) and Kim et al. (2015b). Kim et al. (2021) also appeared to be relatively influential. Random-effect outlier analysis identified Kim et al. (2015b), Malekzadeh et al. (2010), and Tan et al. (2018) as influential outlier studies. After excluding these studies, the pooled treatment failure proportion was 14.6 % (95 % CI, 10.4 %–20.2 %), and heterogeneity decreased to $I^{2}=29.4$ %.

Publication bias was assessed for treatment failure as at least 10 studies were available. Funnel plot inspection suggested asymmetry (Fig. S6), confirmed by Egger's test (intercept -2.197 , p<0.001 ). Trim-and-fill analysis imputed eight potentially missing studies, increasing the pooled failure rate to 25.58 % (95 % CI, 19 %–32 %), indicating that the unadjusted estimate may have underestimated true failure.

Leave-one-out analysis demonstrated overall stability of the pooled estimate, ranging from 16.34 % after exclusion of Tan et al. (2018) to 18.40 % after exclusion of Wang et al. (2018).

Subgroup analysis by treatment strategy (Fig. S7) showed significant differences in failure rates ( p=0.01 ): 29.4 % (95 % CI, 20.3 %–40.6 %; $I^{2}=59.7$ %) for DAIR, 14.0 % (95 % CI, 7.7 %–24.1 %; $I^{2}=34.3$ %) for two-stage exchange, and 13.8 % (95 % CI, 8.8 %–21.1 %; $I^{2}=0$ %) for one-stage exchange arthroplasty.

#### 3.4.3 Mortality

**Figure 4:**
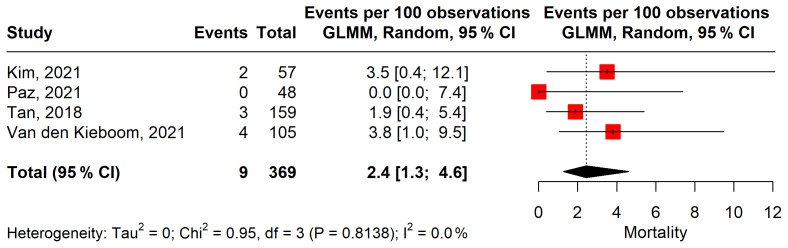
Forest plot of pooled overall mortality.

A meta-analysis of four studies including 369 patients and nine mortality events showed a pooled mortality rate of 2.4 % (95 % CI, 1.3–4.6) using a random-effect model (Fig. 4). No significant between-study heterogeneity was observed ( $\tau^{2}=0$ , $I^{2}=0.0$ %, p=0.81 ). Mortality remained consistently low across studies, and one study reported no deaths.

Influence analysis (Fig. S8) did not identify any outlier studies under either fixed- or random-effect models. Although diagnostic plots suggested some variation in influence, with van Den Kieboom et al. (2021) appearing relatively more prominent, no single study materially affected the pooled estimate.

Leave-one-out analysis further confirmed robustness, with pooled mortality ranging from 1.8 % to 2.8 % and no heterogeneity observed in any iteration ( $I^{2}=0$ %). Publication bias was not assessed due to the small number of included studies ( <10 ).

#### 3.4.4 Meta-regression analysis

Meta-regression analyses were conducted to assess whether follow-up duration and publication year explained between-study heterogeneity in treatment success and failure (Table S2).

For treatment success, neither follow-up duration ( p=0.6265 ) nor publication year ( p=0.4275 ) was significantly associated with pooled estimates, and substantial residual heterogeneity remained ( $I^{2}=73.9$ % and 73.2 %, respectively). Similarly, for treatment failure, no significant associations were observed for follow-up duration ( p=0.9939 ) or publication year ( p=0.5257 ), with persistently high residual heterogeneity ( $I^{2}=71.6$ % and 72.6 %, respectively).

Overall, these results indicate that the evaluated study-level factors did not account for the observed heterogeneity in treatment outcomes.

### 3.5 Quality assessment

Quality assessment using the NOS showed scores ranging from 5 to 9, indicating overall moderate to high methodological quality. Among the 27 included studies, 2 scored 9, 7 scored 8, 9 scored 7, 6 scored 6, and 3 scored 5 (Table S1). Most studies performed well in terms of exposure ascertainment and outcome assessment, while lower scores were mainly due to limitations in cohort comparability, selection of the non-exposed cohort, confirmation of the absence of an outcome at baseline, and adequacy of follow-up.

## 4 Discussion

This systematic review and meta-analysis showed that CN-PJI is generally associated with favorable outcomes, with pooled treatment success, failure, and mortality proportions of 83.6 %, 17.2 %, and 2.4 %, respectively. Sensitivity analyses supported the stability of these findings despite several influential studies contributing to heterogeneity. Subgroup analyses showed significant differences by treatment strategy and joint type: DAIR was associated with lower success and higher failure, whereas one-stage and two-stage exchange arthroplasty showed better outcomes. Hip CN-PJI also showed higher treatment success than knee CN-PJI. Although funnel plot asymmetry and Egger's test suggested possible publication bias for success and failure, the overall evidence suggests that CN-PJI does not necessarily confer a poor prognosis, particularly when managed with exchange arthroplasty.

Surgical management of CN-PJI generally follows the same principles as culture-positive PJI. Previous studies have shown that two-stage exchange is the most used approach and is often considered to be the standard treatment, whereas DAIR is usually reserved for selected acute cases with short symptom duration, a stable prosthesis, and no sinus tract (Patel, 2023). In our systematic review, two-stage exchange was also performed more often than one-stage exchange, which is consistent with prior reports (Patel, 2023). Although our subgroup analysis showed similar success rates for one-stage and two-stage exchange, this finding should be interpreted with caution.

This cautious interpretation is also supported by recent literature on culture-positive and joint-specific PJI populations. Similarly, previous studies comparing one-stage and two-stage exchange in knee, hip, and shoulder PJI have reported comparable outcomes while emphasizing the influence of selection bias (Lazic et al., 2021; Kunutsor et al., 2018; Belay et al., 2020). Similarly, Thakrar et al. (2019) reported that favorable outcomes with one-stage exchange are mainly observed in carefully selected patients without significant host compromise, severe soft-tissue or bone defects, or resistant or atypical organisms while also acknowledging the inherent selection bias in observational studies. Taken together, these findings suggest that comparable results with one-stage exchange should not be interpreted as being equivalent to those with two-stage exchange but rather should be interpreted as evidence that one-stage exchange can achieve good outcomes in appropriately selected patients.

Antimicrobial management in CN-PJI was highly heterogeneous and often incompletely reported. When available, regimens were largely individualized rather than protocol-driven. Vancomycin-based therapy was most common, with frequent use of cephalosporins and fluoroquinolones, including oral step-down treatment. This variability likely reflects limited evidence guiding empirical therapy. Broad-spectrum use may also increase toxicity and other adverse events, highlighting the need for better diagnostics and antimicrobial stewardship. Prior evidence suggests that empirical regimens should primarily cover gram-positive organisms, especially staphylococci, with additional gram-negative coverage (Kalbian et al., 2020). This approach is reasonable because *staphylococci* are common causes of PJI, and resistant or polymicrobial infections may also occur, particularly in acute cases (Kim et al., 2022). Previous studies have suggested that vancomycin plus gram-negative coverage may be preferred for early PJI, whereas beta-lactams or trimethoprim-sulfamethoxazole may be sufficient for late or chronic PJI. However, treatment should be adjusted if a pathogen is subsequently identified by culture or molecular testing (Guo et al., 2026; Kim et al., 2022). In our review, vancomycin, cephalosporins, fluoroquinolones, rifampin, and other broad-spectrum combination regimens were commonly reported, but no standard antibiotic regimen was identified across studies. Prior literature suggests that treatment is often prolonged, with 12 weeks generally being preferred over 6 weeks, particularly in DAIR-managed cases, and that oral agents with good bioavailability are frequently used after an initial intravenous phase. Overall, antibiotic therapy for CN-PJI remains largely individualized. The substantial heterogeneity across studies likely reflects limited high-quality evidence, variability in patient and infection characteristics, and the lack of clear guideline-based recommendations.

From a clinical perspective, the most important finding of this review is that culture-negative PJI was associated with a relatively high pooled treatment success rate despite the absence of pathogen identification. This observation suggests that favorable outcomes remain achievable when treatment decisions are guided by established surgical principles and empiric antimicrobial therapy. While exchange arthroplasty demonstrated higher pooled success rates than DAIR, these findings should not be interpreted as evidence that culture status alone should determine surgical management as treatment selection is strongly influenced by infection chronicity, implant stability, host factors, and other clinical considerations. Instead, our findings highlight the need for future studies aimed at identifying which patients with culture-negative PJI are most likely to benefit from specific surgical approaches and empiric antimicrobial strategies.

This study has several strengths. It provides an updated and comprehensive synthesis of the available evidence on CN-PJI and includes more studies and patients than prior reviews. The meta-analysis allowed pooled estimates of treatment success, failure, and mortality, and sensitivity, influence, and subgroup analyses supported a more detailed interpretation of outcomes by treatment strategy and joint type.

However, several limitations should be noted. Most included studies were retrospective, with inherent risks of selection bias, confounding, and incomplete reporting. There was considerable clinical and methodological heterogeneity, including differences in patient selection, diagnostic approaches, surgical strategies, antimicrobial regimens, and follow-up duration. Inconsistent definitions of treatment success, failure, and reinfection further limited comparability. Despite efforts by Diaz-Ledezma et al. (2013), the Musculoskeletal Infection Society (MSIS) (2019) (Fillingham et al., 2019), and Johns et al. (2022) to establish standardized definitions of success and failure in periprosthetic joint infection (PJI) management, considerable variability remains across the literature. Many studies continue to use institution-specific or study-specific outcome definitions, contributing to substantial heterogeneity among reported results. Although this heterogeneity is an important limitation, it cannot be fully addressed at the current stage due to the lack of uniform outcome reporting across the included studies. We have previously recognized the challenges posed by this variability and, in our earlier work, proposed a definition of treatment success and failure based on the most clinically relevant and widely accepted criteria. Nevertheless, the absence of a universal adoption of standardized outcome measures continues to contribute to residual heterogeneity across studies (Alavi et al., 2026). Variability in diagnostic methods may also have affected CN case classification. In addition, potential publication bias in reported success and failure rates warrants cautious interpretation of pooled results.

Our findings underscore the need for prospective, multi-center studies with standardized definitions of CN-PJI, treatment success, failure, and reinfection, as well as clearer reporting of antimicrobial regimens and surgical selection criteria. Such studies are essential to better define optimal management and to reduce uncertainty arising from heterogeneity and selection bias in the current literature.

## 5 Conclusion

In conclusion, this systematic review and meta-analysis suggests that CN-PJI is generally associated with favorable outcomes, particularly when managed with exchange arthroplasty. DAIR was associated with lower success and higher failure, and outcomes appeared to be better in hip than in knee PJI. However, interpretation remains limited by heterogeneity in diagnostic methods, outcome definitions, and study design. Further prospective studies with standardized definitions and reporting are needed to better guide management.

## Supplement

10.5194/jbji-11-413-2026-supplement The supplement related to this article is available online at https://doi.org/10.5194/jbji-11-413-2026-supplement.

## Data Availability

All data used in this study were obtained from previously published articles cited in the paper. The extracted dataset supporting the findings of this study is available from the corresponding author upon reasonable request.
