# Supplementary material for: Management and outcomes of culture-negative periprosthetic joint infection: a systematic review and meta-analysis
Source: J Bone Jt Infect. 2026 Jul 14;11(4):413–30. doi: 10.5194/jbji-11-413-2026 (PMC13448843; doi:10.5194/jbji-11-413-2026)
Supplement: Supplementary file 1 [file jbji-11-413-2026-supplement.pdf]

Supplement of J. Bone Joint Infect., 11, 413–430, 2026
https://doi.org/10.5194/jbji-11-413-2026-supplement
© Author(s) 2026. CC BY 4.0 License.

Supplement of

Management and outcomes of culture-negative periprosthetic joint infection: a systematic review and meta-analysis

Farzad Pourghazi et al.

Correspondence to: Elie F. Berbari (berbari.elie@mayo.edu)

The copyright of individual parts of the supplement might differ from the article licence.

Search Strategy:

Search Strategies

OVID

Database(s): Ovid MEDLINE(R) 1946 to Present and Epub Ahead of Print, In-Process & Other Non-Indexed Citations and Ovid MEDLINE(R) Daily, EBM Reviews - Cochrane Central Register of Controlled Trials June 2025, EBM Reviews - Cochrane Database of Systematic Reviews 2005 to July 2, 2025, Embase 1974 to 2025 July 03
Search Strategy:

#	Searches
1	(Prosthesis-Related Infections/ or prosthesis infection/) and (joint* or hip* or knee* or shoulder* or elbow*).ti,ab.
2	(pji or (periprosthe* adj1 infect*) or (infected adj2 joint adj1 arthroplast*) or (prosthe* adj2 infect*) or (arthroplast* adj2 infect*) or (joint* adj1 infect*) or infected-prosthetic-arthroplast* or joint-replacement-infection* or infected-total-joint-arthroplast* or peri-implant-infection* or prosthesis-related infection* or infected-joint-replacement*).ti.
3	1 or 2
4	exp High-Throughput Nucleotide Sequencing/
5	exp *Microbiological Techniques/ or *microbiological examination/
6	(mi or di).fs.
7	"next-generation-sequencing".ti.
8	(diagnos* or test* or lab or laboratory or sonication or "sonicate-fluid" or "polymerase-chain-reaction" or "16S-RNA" or metagenomic* or "synovial-fluid-culture").ti,ab.
9	or/4-8
10	(negativ* or identify* or unidentified or inconclusive or steril* or accurate or accuracy).ti,ab.
11	Prosthesis-Related Infections/di or exp prosthesis infection/di
12	10 or 11
13	9 and 12
14	("culture-negative" or CN-PJI).ti,ab,hw,kf,kw. or (sterile adj2 culture*).ti,ab. or (culture* adj2 unidentified).ti,ab. or (staph* adj2 negative).ti,ab.
15	13 or 14
16	3 and 15
17	from 16 keep 1-6000
18	remove duplicates from 17
19	16 not 17
20	remove duplicates from 19
21	18 or 20

SCOPUS

1 TITLE(pji) OR TITLE-ABS-KEY (periprosthe* W/1 infect*) OR TITLE-ABS-KEY (infect* W/2 joint*) OR TITLE-ABS-KEY (prosthe* W/2 infect*) OR TITLE-ABS-KEY (arthroplast* W/2 infect*) OR TITLE-ABS-KEY (joint* W/1 infect*) OR TITLE-ABS-KEY (infected-prosthetic-arthroplast* OR joint-replacement-infection* OR infected-total-joint-arthroplast* OR peri-implant-infection* OR prosthesis-related infection* OR infected-joint-replacement*)

2 (TITLE-ABS-KEY ("culture-negative" or CN-PJI) OR TITLE-ABS-KEY (sterile W/2 culture*) or TITLE-ABS-KEY (culture* W/2 unidentified) or TITLE-ABS-KEY (staph* W/2 negative)) OR (TITLE (negativ* OR identify* OR unidentified OR inconclusive OR steril* OR accurate OR accuracy))

3 1 and 2

4 INDEX (embase) OR INDEX (medline) OR PMID (0* OR 1* OR 2* OR 3* OR 4* OR 5* OR 6* OR 7* OR 8* OR 9*)

5 3 NOT 4

Extracted Variables:

Extracted variables included study design and setting, sample size, affected joint, type and duration of antimicrobial therapy, surgical intervention (DAIR, one-stage exchange, two-stage exchange, resection arthroplasty, or arthrodesis), and clinical outcomes, including treatment success and failure and mortality.

Eligibility criteria

The inclusion criteria were as follows: (a) studies including adult participants (≥18 years); (b) studies including patients with CN-PJI, either as the full study population or as a mixed cohort if outcomes for the CN group were reported separately; (c) studies reporting medical and/or surgical treatment strategies for CN-PJI with clinical outcomes; (d) English-language publications; and (e) studies including at least five patients with CN-PJI. CN-PJI was defined according to each original study. Exclusion criteria were: (a) pediatric studies; (b) reviews, systematic reviews, meta-analyses, study protocols, or preprints; and (c) non-human studies. The primary objective of this review was to summarize the reported outcomes of different treatment strategies for CN-PJI.

Reference lists of all included studies:

Berbari, E. F., Marculescu, C., Sia, I., Lahr, B. D., Hanssen, A. D., Steckelberg, J. M., Gullerud, R., and Osmon, D. R.: Culture-negative prosthetic joint infection, Clinical infectious diseases, 45, 1113-1119, 2007.

Browning, S., Manning, L., Metcalf, S., Paterson, D. L., Robinson, J. O., Clark, B., and Davis, J. S.: Characteristics and outcomes of culture-negative prosthetic joint infections from the Prosthetic Joint Infection in Australia and New Zealand Observational (PIANO) cohort study, Journal of Bone and Joint Infection, 7, 203-211, 2022.

Choi, H.-R., Kwon, Y.-M., Freiberg, A. A., Nelson, S. B., and Malchau, H.: Periprosthetic joint infection with negative culture results: clinical characteristics and treatment outcome, The Journal of arthroplasty, 28, 899-903, 2013.

Garabano, G., Gessara, A. M., Pesciallo, C. A., Martinez, J., and Del Sel, H.: Culture-negative peri-prosthetic joint infection after total hip arthroplasty treatment protocol and outcomes in acute and chronic cases, Archives of Bone and Joint Surgery, 10, 806, 2022.

Ibrahim, M., Twaij, H., and Haddad, F.: Two-stage revision for the culture-negative infected total hip arthroplasty: a comparative study, The bone & joint journal, 100, 3-8, 2018.

Ji, B., Li, G., Zhang, X., Wang, Y., Mu, W., and Cao, L.: Effective treatment of single-stage revision using intra-articular antibiotic infusion for culture-negative prosthetic joint infection: a comparative study, The bone & joint journal, 102, 336-344, 2020.

Kang, J.-S., Shin, E.-H., Roh, T.-H., Na, Y., Moon, K. H., and Park, J.-H.: Long-term clinical outcome of two-stage revision surgery for infected hip arthroplasty using cement spacer: Culture negative versus culture positive, Journal of Orthopaedic Surgery, 26, 2309499017754095, 2018.

Kim, C.-W., Lee, C.-R., Park, D.-H., Kim, D.-Y., and Kim, J.-W.: Clinical outcomes of two-stage revision for chronic periprosthetic joint infection of the knee: culture-negative versus culture-positive, Knee Surgery & Related Research, 33, 28, 2021.

Kim, Y.-H., Park, J.-W., Kim, J.-S., and Kim, D.-J.: The outcome of infected total knee arthroplasty: culture-positive versus culture-negative, Archives of orthopaedic and trauma surgery, 135, 1459-1467, 2015a.

Kim, Y.-H., Kulkarni, S. S., Park, J.-W., Kim, J.-S., Oh, H.-K., and Rastogi, D.: Comparison of infection control rates and clinical outcomes in culture-positive and culture-negative infected total-knee arthroplasty, Journal of orthopaedics, 12, S37-S43, 2015b.

Li, H., Ni, M., Li, X., Zhang, Q., Li, X., and Chen, J.: Two-stage revisions for culture-negative infected total knee arthroplasties: A five-year outcome in comparison with one-stage and two-stage revisions for culture-positive cases, Journal of Orthopaedic Science, 22, 306-312, 2017.
Lin, L., Li, J., Zhang, C., Li, J., Wu, B., Huang, Z., Lv, J., Liu, M., Li, W., and Zhang, W.: Comprehensive analysis of culture-negative periprosthetic joint infection with metagenomic next-generation sequencing, Frontiers in Cellular and Infection Microbiology, 15, 1564488, 2025.
Lu, H., Wang, W., Xu, H., Zhou, K., and Zhou, Z.: Efficacy and safety of two-stage revision for patients with culture-negative versus culture-positive periprosthetic joint infection: a single-center retrospective study, BMC Musculoskeletal Disorders, 25, 160, 2024.
Malekzadeh, D., Osmon, D. R., Lahr, B. D., Hanssen, A. D., and Berbari, E. F.: Prior use of antimicrobial therapy is a risk factor for culture-negative prosthetic joint infection, Clinical Orthopaedics and Related Research®, 468, 2039-2045, 2010.
Paz, Z., Zhu, C., Lieber, S. B., Fowler, M. L., and Shmerling, R. H.: Presentation and outcomes of peri-prosthetic joint infection: A comparison of culture-positive and culture-negative disease, Surgical Infections, 22, 828-835, 2021.
Ronan, E. M., Ruff, G., Ashkenazi, I., Raymond, H., Cardillo, C., Villa, J. C., Schwarzkopf, R., and Aggarwal, V. K.: The impact of culture negativity on the outcomes of revision total knee arthroplasty for chronic PJI, Microorganisms, 12, 1384, 2024.
Santoso, A., Park, K.-S., Shin, Y.-R., Yang, H.-Y., Choi, I.-S., and Yoon, T.-R.: Two-stage revision for periprosthetic joint infection of the hip: Culture-negative versus culture-positive infection, Journal of Orthopaedics, 15, 391-395, 2018.
Soundarrajan, D., Rajkumar, N., Dhanasekararaja, P., Rithika, S., and Rajasekaran, S.: A comparison of outcomes of culture positive and culture negative acute knee prosthetic joint infection following debridement, antibiotics and implant retention (DAIR), European Journal of Orthopaedic Surgery & Traumatology, 33, 2375-2383, 2023.
Tan, T. L., Kheir, M. M., Shohat, N., Tan, D. D., Kheir, M., Chen, C., and Parvizi, J.: Culture-negative periprosthetic joint infection: an update on what to expect, JBJS Open Access, 3, e0060, 2018.
Tirumala, V., Smith, E., Box, H., van den Kieboom, J., Klemt, C., and Kwon, Y.-M.: Outcome of debridement, antibiotics, and implant retention with modular component exchange in acute culture-negative periprosthetic joint infections, The Journal of Arthroplasty, 36, 1087-1093, 2021.
Vajapey, S. P., Li, D., Lynch, D., and Li, M.: Prosthetic joint infection in culture-negative and alpha-defensin-positive patients versus culture-positive and alpha-defensin-negative patients: a retrospective cohort study of the differences in clinical characteristics and outcomes, Current Orthopaedic Practice, 32, 58-63, 2021.
van Den Kieboom, J., Tirumala, V., Box, H., Oganesyan, R., Klemt, C., and Kwon, Y.-M.: One-stage revision is as effective as two-stage revision for chronic culture-negative periprosthetic joint infection after total hip and knee arthroplasty: a retrospective cohort study, The Bone & Joint Journal, 103, 515-521, 2021.
van Sloten, M., Gómez-Junyent, J., Ferry, T., Rossi, N., Petersdorf, S., Lange, J., Corona, P., Abreu, M. A., Borens, O., and Zlatian, O.: Should all patients with a culture-negative periprosthetic joint infection be treated with antibiotics?: a multicentre observational study, The bone & joint journal, 104, 183-188, 2022.
Wang, J., Wang, Q., Shen, H., and Zhang, X.: Comparable outcome of culture-negative and culture-positive periprosthetic hip joint infection for patients undergoing two-stage revision, International orthopaedics, 42, 469-477, 2018.
Watanabe, S., Kobayashi, N., Tomoyama, A., Choe, H., Yamazaki, E., and Inaba, Y.: Clinical characteristics and risk factors for culture-negative periprosthetic joint infections, Journal of orthopaedic surgery and research, 16, 292, 2021.
Xu, Z., Huang, C., Lin, Y., Chen, Y., Fang, X., Huang, Z., Zhang, C., Zhang, Z., and Zhang, W.: Clinical Outcomes of Culture-Negative and Culture-Positive Periprosthetic Joint Infection: Similar Success Rate, Different Incidence of Complications, Orthopaedic Surgery, 14, 1420-1427, 2022.
Zanna, L., Sangaletti, R., Lausmann, C., Gehrke, T., and Citak, M.: Successful eradication rate following one-stage septic knee and hip exchange in selected pre-operative culture-negative periprosthetic joint infections, International orthopaedics, 47, 659-666, 2023.

Table S1. Quality Assessment of cohort studies using Newcastle–Ottawa Scale (NOS)

	**Selection**				**Comparability**	**Outcome**			
Author, Year	*1) Representativeness of the Exposed Cohort	*2) Selection of the Non-Exposed Cohort	*3) Ascertainment of Exposure	*4) Demonstration That Outcome of Interest Was Not Present at Start of Study	**1) Comparability of Cohorts on the Basis of the Design or Analysis	*1) Assessment of Outcome	*2)Was Follow-Up Long Enough for Outcomes to Occur	*3) Adequacy of Follow Up of Cohorts	Total score
Berbari, 2007	*	-	*	-	-	*	*	-	5
Browning, 2022	*	*	*	**	**	*	*	-	8
Choi, 2013	*	*	*	-	-	*	*	-	6
Garabano,2022	*	-	*	-	-	*	*	*	6
Ibrahim,2018	*	*	*	*	*	*	*	*	8
Ji, 2020	*	*	*	*	*	*	*	*	8
Kang, 2018	*	*	*	*	*	*	*	*	8
Kim, 2021	*	*	*	*	*	*	*	-	7
Kim, 2015	*	*	*	-	-	*	*	*	7
Kim, 2015	*	*	*	-	-	*	*	*	7
Li, 2017	*	*	*	-	-	*	*	*	7
Lin, 2025	*	*	*	-	-	*	*	*	7
Lu, 2024	*	*	*	*	*	*	*	*	8
Malekzadeh, 2010	*	*	*	**	**	*	*	*	9
Paz, 2021	*	*	*	-	-	*	*	-	6
Ronan, 2024	*	*	*	*	*	*	*	*	8
Santoso, 2018	*	*	*	*	*	*	*	-	7
Soundarrajan,2023	*	*	*	**	**	*	*	*	9
Tan, 2018	*	-	*	-	-	*	*	-	5

Tirumala, 2021	*	*	*	*	*	*	*	*	8
Vajapey, 2021	*	*	*	-	-	*	-	-	5
Van Den Kieboom, 2021	*	*	*	-	-	*	*	-	6
Van Sloten, 2022	*	*	*	-	-	*	*	*	7
Wang, 2018	*	*	*	*	*	*	*	-	7
Watanabe, 2021	*	*	*	-	-	*	*	-	6
Xu, 2022	*	*	*	-	-	*	*	*	7
Zanna, 2023	*	-	*	-	-	*	*	*	6

Table S2. Meta-regression results evaluating publication year and follow-up duration (months) as moderators of pooled outcome estimates (success, failure), reporting residual heterogeneity (τ^2, I^2, H^2) and the moderator test (QM p-value).

**Outcome**	**Moderator**	**k**	**Residual τ^2**	**Residual I^2 (%)**	**Residual H^2**	**P value for moderator (QM)**
Treatment success	Follow-up duration (months)	23	0.4160	73.90	3.83	0.6265
Treatment success	Publication year	23	0.3810	73.16	3.73	0.4275
Treatment failure	Follow-up duration (months)	21	0.3643	71.58	3.52	0.9939
Treatment failure	Publication year	21	0.3582	72.57	3.65	0.5257

Supplementary Figures

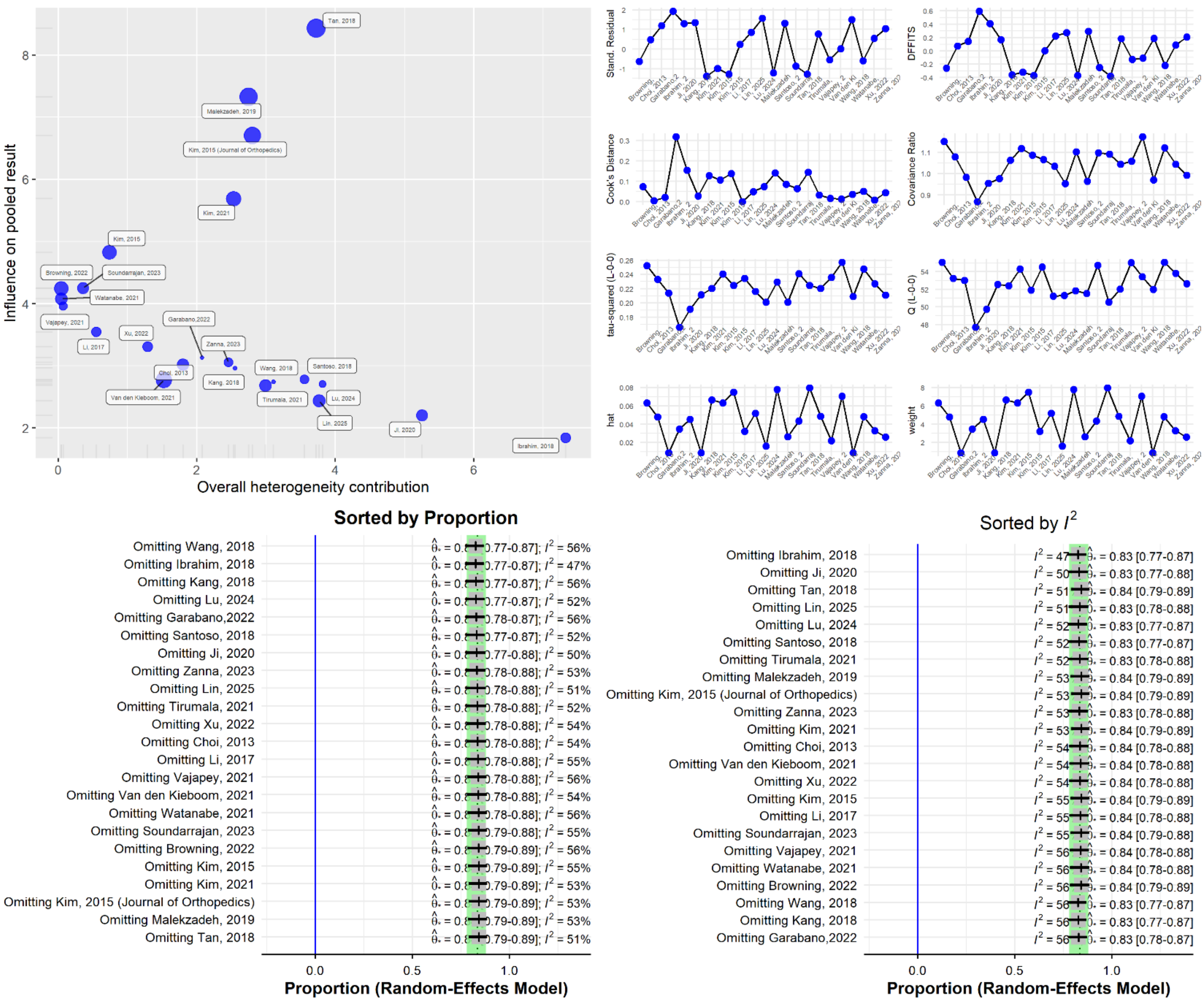

Fig. S1. Influence analysis and leave-one-out sensitivity analysis for overall treatment success.

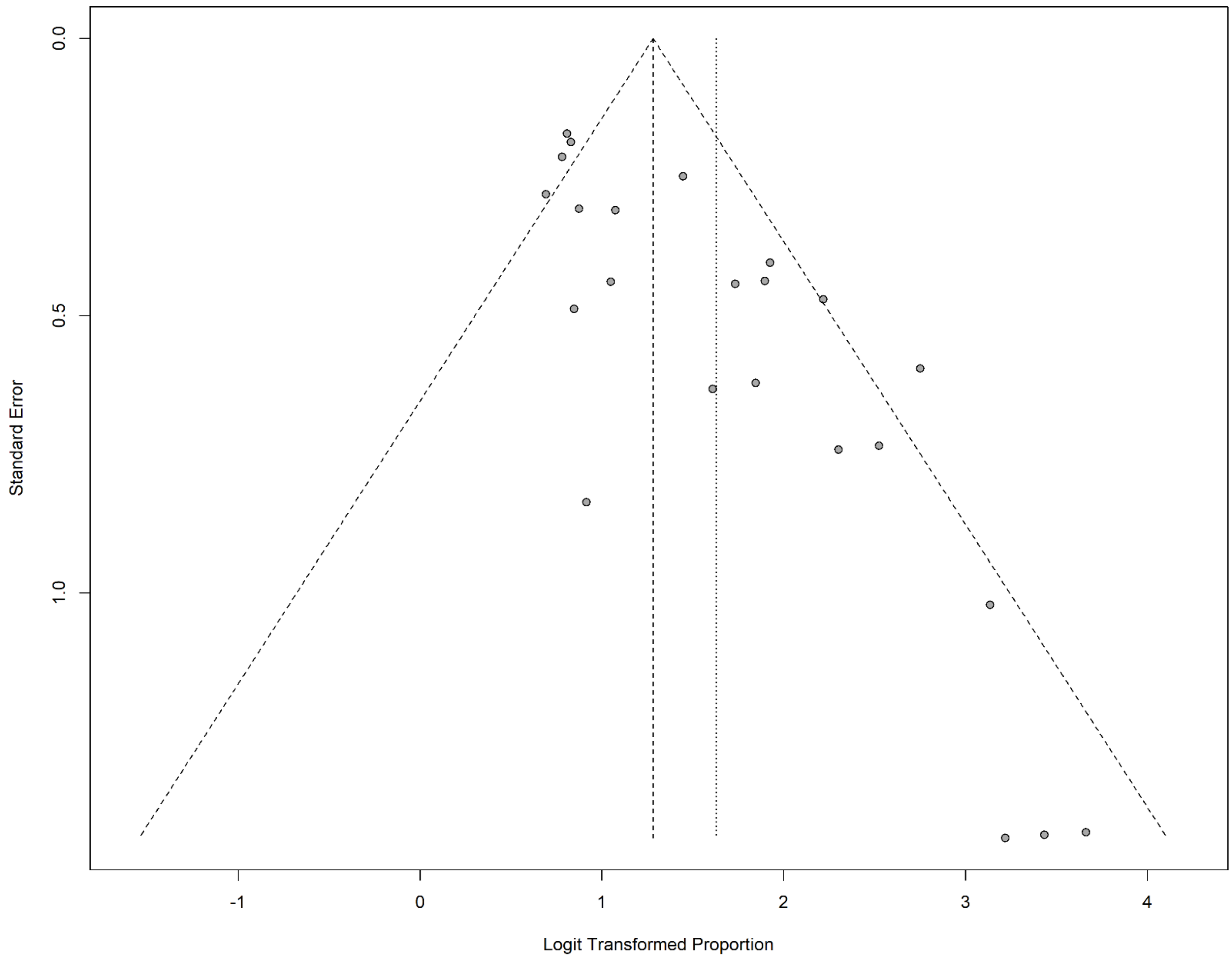

Fig. S2. Funnel plot for overall treatment success. Visual inspection of the funnel plot suggests asymmetry, with a relative paucity of smaller studies on the left side of the plot and several small studies reporting higher success rates on the right, raising concern for small-study effects or publication bias. This was supported by Egger's test ($p < 0.0001$).

Study or Subgroup	Events	Total	Events per 100 observations GLMM, Random, 95% CI
**Intervention = DAIR**			
Browning, 2022	23	29	79.3 [60.3; 92.0]
Garabano,2022	8	8	100.0 [63.1; 100.0]
Kim, 2015	17	28	60.7 [40.6; 78.5]
Kim, 2015 (Journal of Orthopedics)	32	56	57.1 [43.2; 70.3]
Soundarrajan, 2023	14	20	70.0 [45.7; 88.1]
Tan, 2018	15	27	55.6 [35.3; 74.5]
Tirumala, 2021	40	46	87.0 [73.7; 95.1]
Xu, 2022	6	7	85.7 [42.1; 99.6]
**Total (95% CI)**	**155**	**221**	**73.2 [61.2; 82.5]**
Heterogeneity: $Tau^2 = 0.3218$; $Chi^2 = 14.88$, df = 7 (P = 0.0376); $I^2 = 52.9\%$			
**Intervention = Two Stage Exchange**			
Browning, 2022	8	11	72.7 [39.0; 94.0]
Ibrahim, 2018	47	50	94.0 [83.5; 98.7]
Kang, 2018	15	15	100.0 [78.2; 100.0]
Kim, 2021	38	57	66.7 [52.9; 78.6]
Kim, 2015	19	23	82.6 [61.2; 95.0]
Kim, 2015 (Journal of Orthopedics)	38	46	82.6 [68.6; 92.2]
Li, 2017	15	18	83.3 [58.6; 96.4]
Lu, 2024	23	24	95.8 [78.9; 99.9]
Santoso, 2018	25	27	92.6 [75.7; 99.1]
Tan, 2018	84	118	71.2 [62.1; 79.2]
Van Den Kieboom, 2021	60	75	80.0 [69.2; 88.4]
Wang, 2018	19	19	100.0 [82.4; 100.0]
Xu, 2022	9	9	100.0 [66.4; 100.0]
**Total (95% CI)**	**400**	**492**	**87.4 [79.2; 92.7]**
Heterogeneity: $Tau^2 = 0.6155$; $Chi^2 = 21.18$, df = 12 (P = 0.0478); $I^2 = 43.4\%$			
**Intervention = One Stage Exchange**			
Ji, 2020	46	51	90.2 [78.6; 96.7]
Tan, 2018	11	14	78.6 [49.2; 95.3]
Van Den Kieboom, 2021	25	30	83.3 [65.3; 94.4]
Xu, 2022	4	6	66.7 [22.3; 95.7]
Zanna, 2023	20	22	90.9 [70.8; 98.9]
**Total (95% CI)**	**106**	**123**	**86.2 [78.9; 91.2]**
Heterogeneity: $Tau^2 = 0$; $Chi^2 = 3.6$, df = 4 (P = 0.4631); $I^2 = 0\%$			

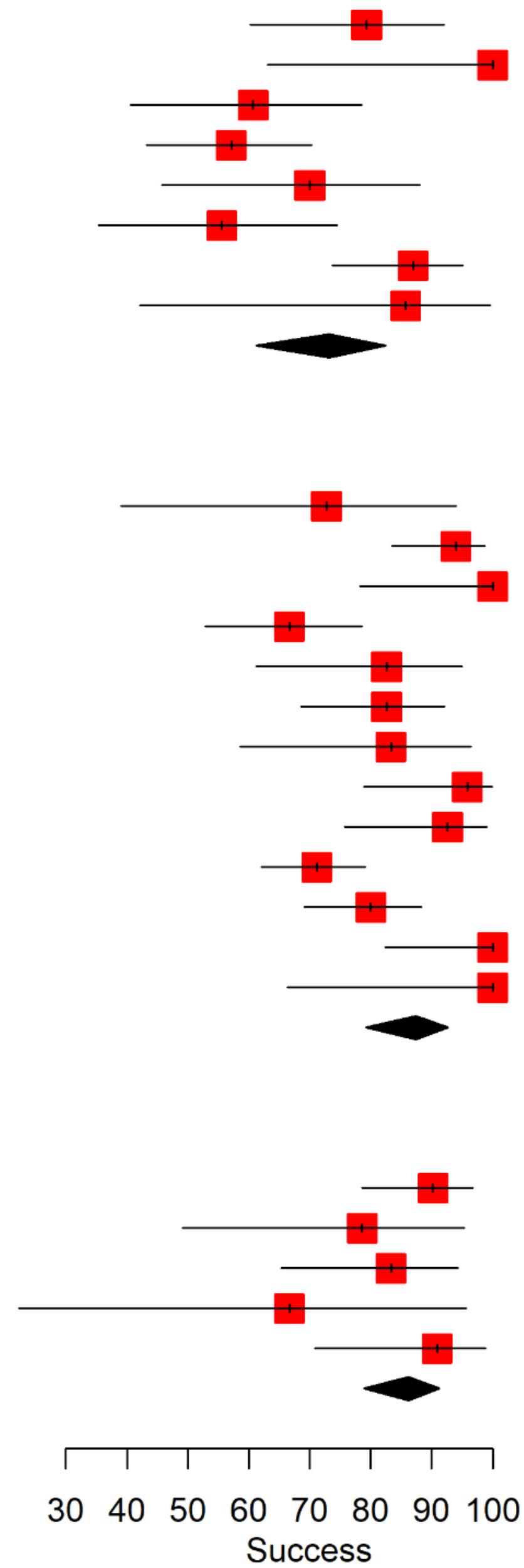

Heterogeneity: $Tau^2 = 0.5437$; $Chi^2 = 53.26$, df = 25 (P = 0.0008); $I^2 = 53.1\%$
Test for subgroup differences: $Chi^2 = 6.55$, df = 2 (P = 0.0378)

Fig. S3. Subgroup meta-analysis of treatment success according to surgical strategy. In the random-effects subgroup analysis, pooled treatment success was 73.2% (95% CI, 61.2%–82.5%; I² = 52.9%) for DAIR, 87.4% (95% CI, 79.2%–92.7%; I² = 43.4%) for two-stage exchange, and 86.2% (95% CI, 78.9%–91.2%; I² = 0%) for one-stage exchange. The difference between treatment-strategy subgroups was statistically significant (p = 0.0378)

Study or Subgroup	Events	Total	Events per 100 observations GLMM, Random, 95% CI
**Joint = Hip**			
Garabano,2022	12	12	100.0 [73.5; 100.0]
Ibrahim, 2018	47	50	94.0 [83.5; 98.7]
Ji, 2020	17	19	89.5 [66.9; 98.7]
Kang, 2018	15	15	100.0 [78.2; 100.0]
Santoso, 2018	25	27	92.6 [75.7; 99.1]
Wang, 2018	19	19	100.0 [82.4; 100.0]
Zanna, 2023	10	12	83.3 [51.6; 97.9]
**Total (95% CI)**	**145**	**154**	**94.2 [89.2; 96.9]**
Heterogeneity: $Tau^2 = 0$; $Chi^2 = 1.5$, df = 6 (P = 0.9594); $I^2 = 0\%$			
**Joint = Knee**			
Ji, 2020	29	32	90.6 [75.0; 98.0]
Kim, 2021	38	57	66.7 [52.9; 78.6]
Kim, 2015	36	51	70.6 [56.2; 82.5]
Kim, 2015 (Journal of Orthopedics)	70	102	68.6 [58.7; 77.5]
Li, 2017	15	18	83.3 [58.6; 96.4]
Soundarrajan, 2023	14	20	70.0 [45.7; 88.1]
Zanna, 2023	10	10	100.0 [69.2; 100.0]
**Total (95% CI)**	**212**	**290**	**75.8 [65.7; 83.6]**
Heterogeneity: $Tau^2 = 0.1274$; $Chi^2 = 7.21$, df = 6 (P = 0.3016); $I^2 = 16.8\%$			

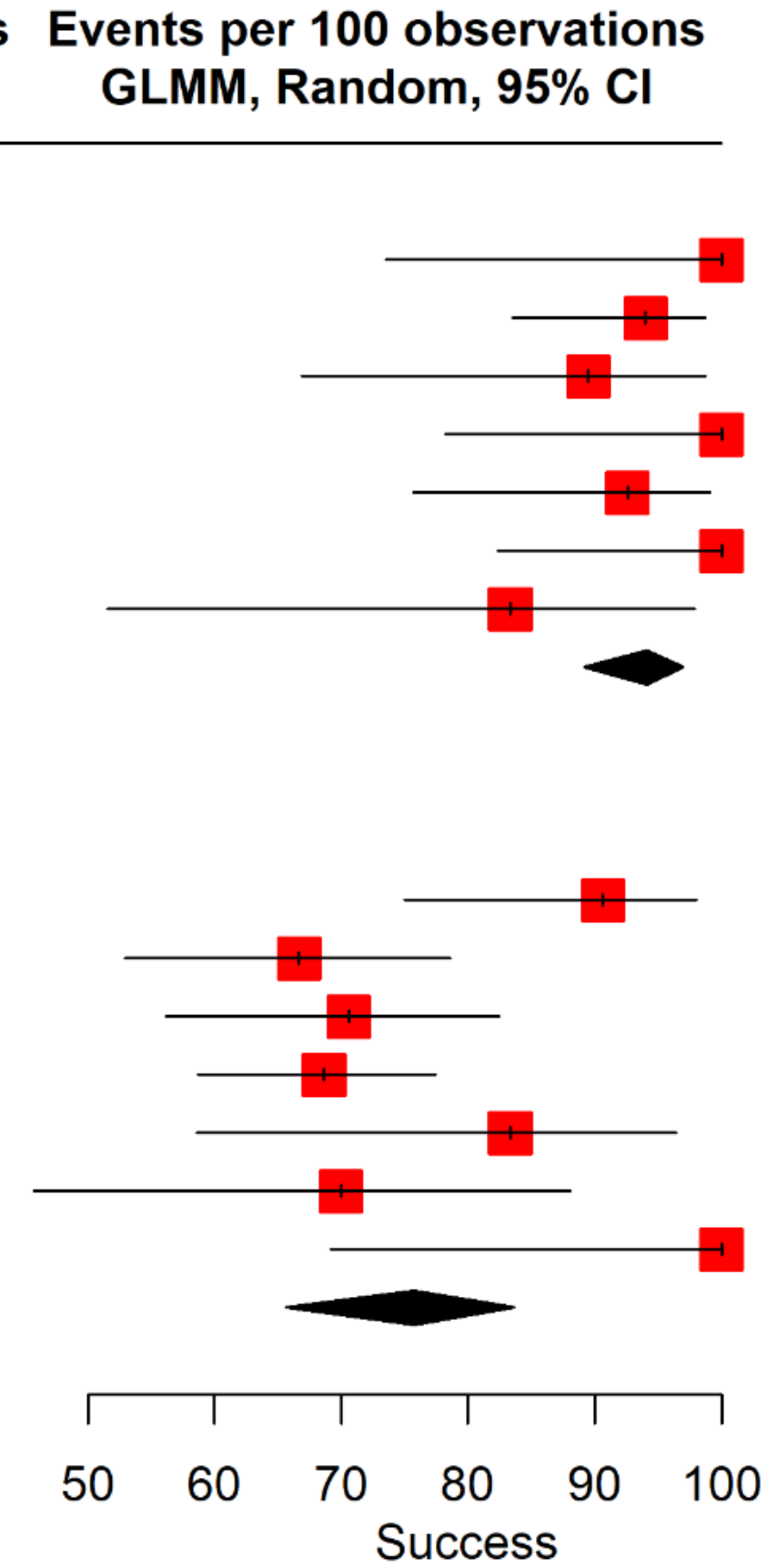

Heterogeneity: $Tau^2 = 0.8899$; $Chi^2 = 23.28$, df = 13 (P = 0.0385); $I^2 = 44.1\%$

Test for subgroup differences: $Chi^2 = 14.84$, df = 1 (P = 0.0001)

Fig. S4. Subgroup meta-analysis of treatment success according to joint type. In the random-effects subgroup analysis, pooled treatment success was significantly higher for hip PJI than for knee PJI, with success rates of 94.2% (95% CI, 89.2%–96.9%; $I^2 = 0\%$) and 75.8% (95% CI, 65.7%–83.6%; $I^2 = 16.8\%$), respectively. The difference between subgroups was statistically significant (p = 0.0001).

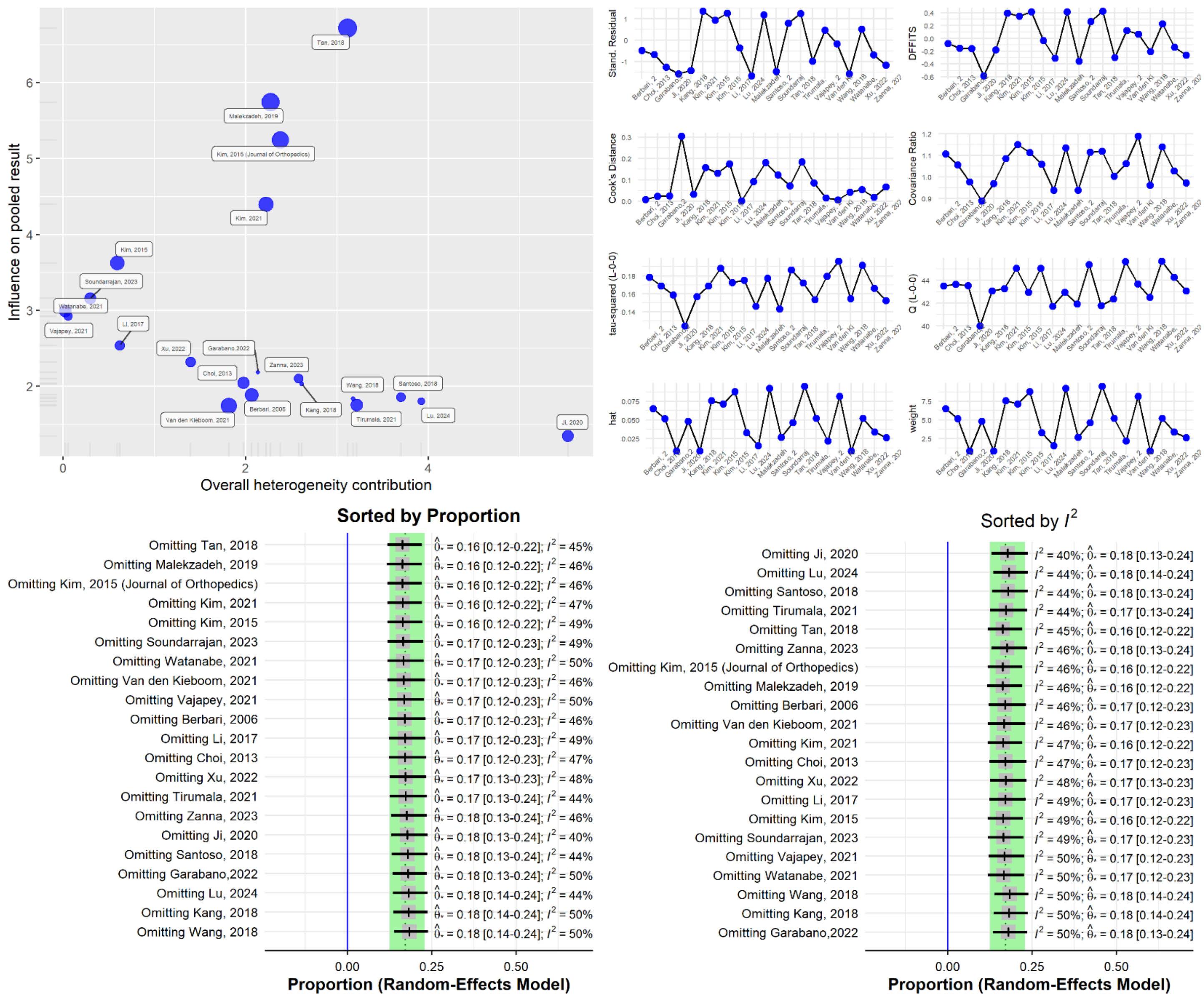

Fig. S5. Influence analysis and leave-one-out sensitivity analysis for treatment failure. Upper panels show study-level influence diagnostics, indicating that Tan, 2018; Malekzadeh, 2019; Kim, 2015 (Journal of Orthopedics); and Kim, 2021 were among the most influential contributors to the pooled treatment failure estimate and between-study heterogeneity.

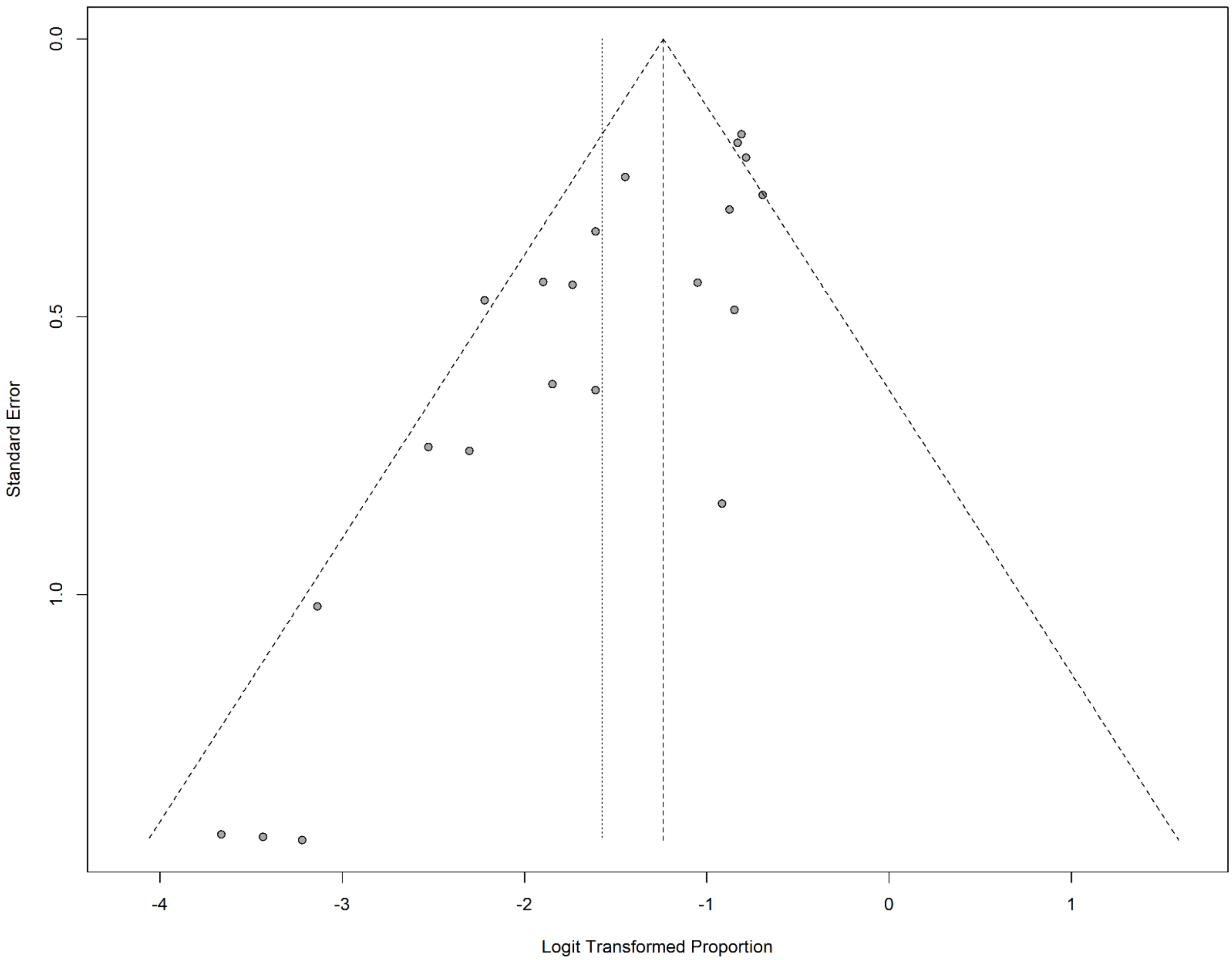

Fig. S6. Funnel plot for treatment failure. Visual inspection of the funnel plot suggests asymmetry, with a relative paucity of smaller studies on the right side of the plot and several small studies with lower reported failure rates on the left, raising concern for small-study effects or publication bias.

Study or Subgroup	Events	Total	Events per 100 observations GLMM, Random, 95% CI
**Intervention = DAIR**			
Browning, 2022	6	29	20.7 [8.0; 39.7]
Kim, 2015	11	28	39.3 [21.5; 59.4]
Kim, 2015 (Journal of Orthopedics)	24	56	42.9 [29.7; 56.8]
Soundarrajan, 2023	6	20	30.0 [11.9; 54.3]
Tan, 2018	12	27	44.4 [25.5; 64.7]
Tirumala, 2021	6	46	13.0 [4.9; 26.3]
Xu, 2022	1	7	14.3 [0.4; 57.9]
**Total (95% CI)**	**66**	**213**	**29.4 [20.3; 40.6]**
Heterogeneity: $Tau^2 = 0.2368$; $Chi^2 = 14.88$, df = 6 (P = 0.0212); $I^2 = 59.7\%$			
**Intervention = Two Stage Exchange**			
Browning, 2022	5	11	45.5 [16.7; 76.6]
Kang, 2018	0	15	0.0 [0.0; 21.8]
Kim, 2021	19	57	33.3 [21.4; 47.1]
Kim, 2015	4	23	17.4 [5.0; 38.8]
Kim, 2015 (Journal of Orthopedics)	8	46	17.4 [7.8; 31.4]
Li, 2017	3	18	16.7 [3.6; 41.4]
Lu, 2024	1	24	4.2 [0.1; 21.1]
Santoso, 2018	2	27	7.4 [0.9; 24.3]
Tan, 2018	34	118	28.8 [20.8; 37.9]
Van Den Kieboom, 2021	15	75	20.0 [11.6; 30.8]
Wang, 2018	0	19	0.0 [0.0; 17.6]
Xu, 2022	0	9	0.0 [0.0; 33.6]
**Total (95% CI)**	**91**	**442**	**14.0 [7.7; 24.1]**
Heterogeneity: $Tau^2 = 0.7361$; $Chi^2 = 16.74$, df = 11 (P = 0.1160); $I^2 = 34.3\%$			
**Intervention = One Stage Exchange**			
Ji, 2020	5	51	9.8 [3.3; 21.4]
Tan, 2018	3	14	21.4 [4.7; 50.8]
Van Den Kieboom, 2021	5	30	16.7 [5.6; 34.7]
Xu, 2022	2	6	33.3 [4.3; 77.7]
Zanna, 2023	2	22	9.1 [1.1; 29.2]
**Total (95% CI)**	**17**	**123**	**13.8 [8.8; 21.1]**
Heterogeneity: $Tau^2 = 0$; $Chi^2 = 3.6$, df = 4 (P = 0.4631); $I^2 = 0\%$			

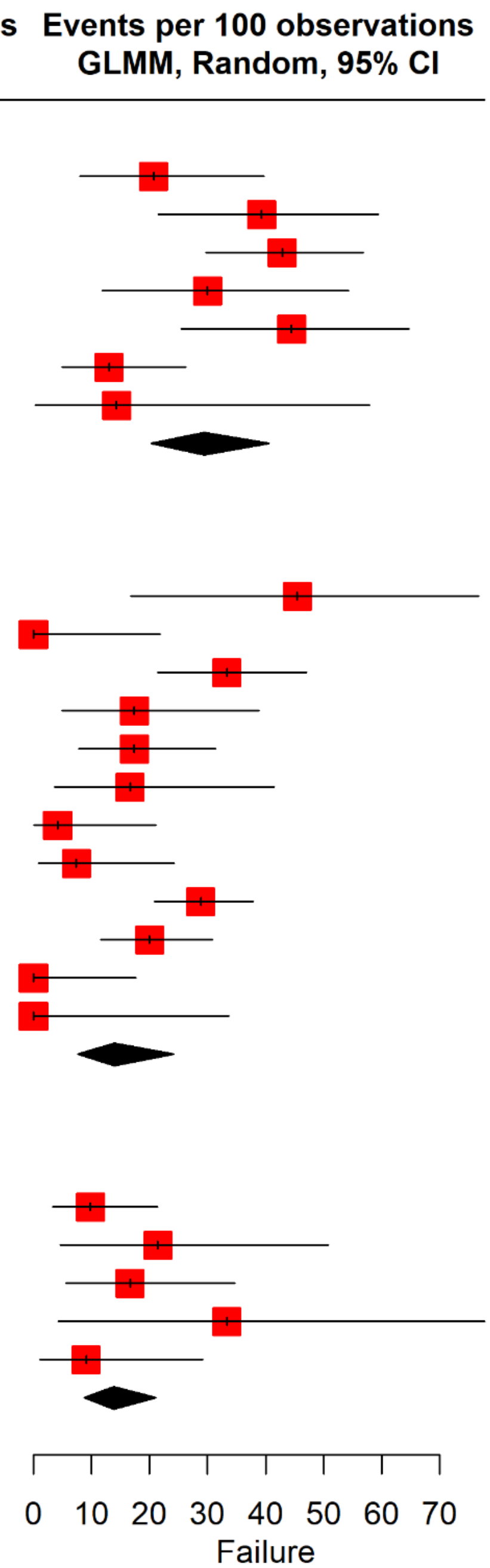

Heterogeneity: $Tau^2 = 0.4971$; $Chi^2 = 47.57$, df = 23 (P = 0.0019); $I^2 = 51.6\%$
Test for subgroup differences: $Chi^2 = 8.51$, df = 2 (P = 0.0142)

Fig. S7. Subgroup meta-analysis of treatment failure according to surgical strategy. In the random-effects subgroup analysis, pooled treatment failure was 29.4% (95% CI, 20.3%–40.6%; $I^2 = 59.7\%$) for DAIR, 14.0% (95% CI, 7.7%–24.1%; $I^2 = 34.3\%$) for two-stage exchange, and 13.8% (95% CI, 8.8%–21.1%; $I^2 = 0\%$) for one-stage exchange. The difference between treatment-strategy subgroups was statistically significant (p = 0.0142).

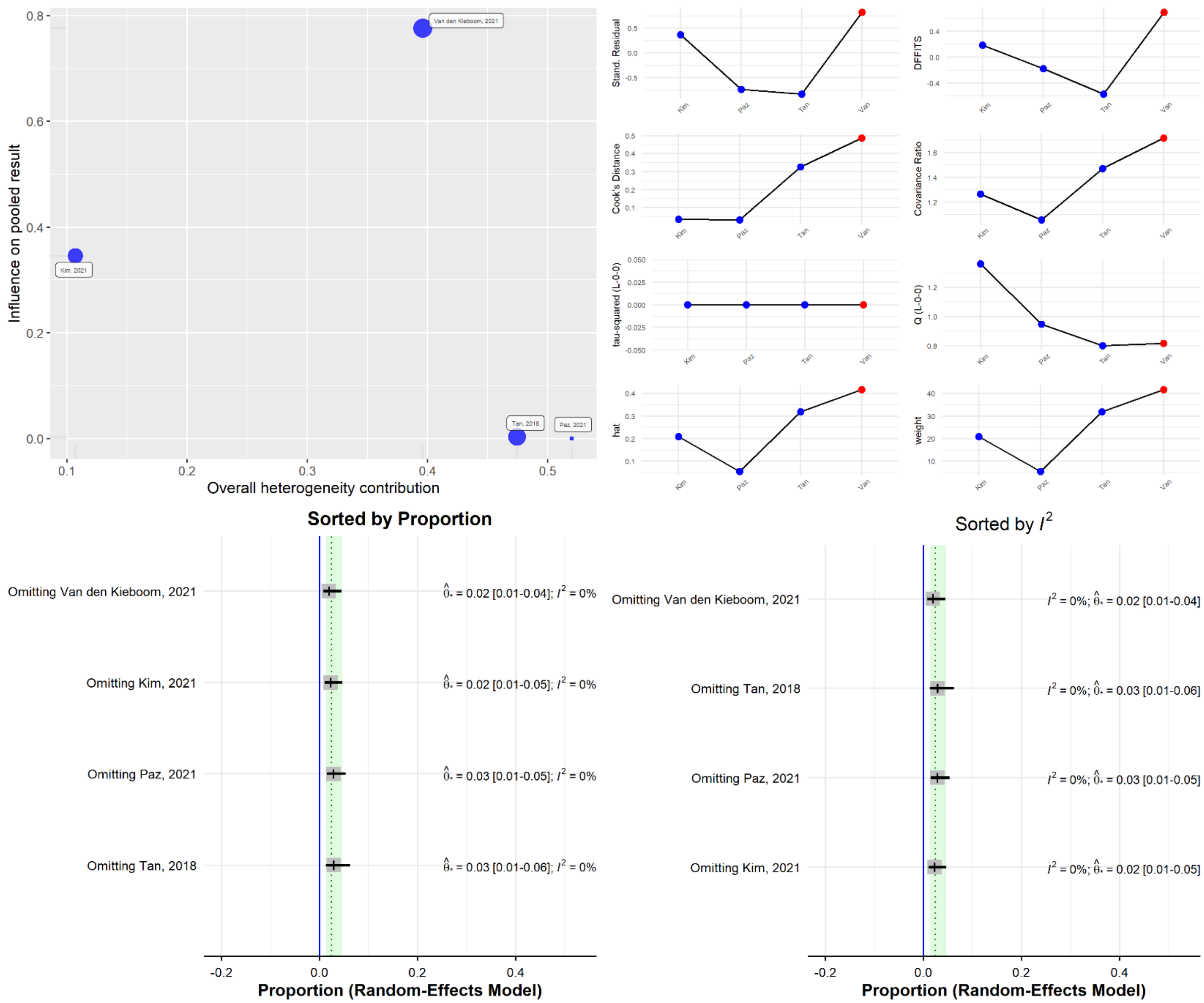

Fig. S8. Influence analysis and leave-one-out sensitivity analysis for mortality. Upper panels show study-level influence diagnostics, with Van den Kieboom, 2021 appearing to contribute most to the pooled mortality estimate and between-study heterogeneity. Lower panels show leave-one-out random-effects analyses, demonstrating that omission of any single study produced only minimal changes in the pooled mortality estimate and did not alter heterogeneity ($I^2 = 0\%$), supporting the stability of the findings.
